# Applicability Evaluation of Full-Reference Image Quality Assessment Methods for Computed Tomography Images

**DOI:** 10.1007/s10278-023-00875-0

**Published:** 2023-08-07

**Authors:** Kohei Ohashi, Yukihiro Nagatani, Makoto Yoshigoe, Kyohei Iwai, Keiko Tsuchiya, Atsunobu Hino, Yukako Kida, Asumi Yamazaki, Takayuki Ishida

**Affiliations:** 1 Division of Health Sciences, Osaka University Graduate School of Medicine, Suita, Japan; 2 Department of Radiology, Shiga University of Medical Science Hospital, Otsu, Japan; 3 Department of Radiology, Omihachiman Community Medical Center, Omihachiman, Japan; 4 Department of Radiology, Nagahama Red Cross Hospital, Nagahama, Japan

**Keywords:** Image quality, FR-IQA, Computed tomography, Objective assessment, Subjective assessment, VIF

## Abstract

Image quality assessments (IQA) are an important task for providing appropriate medical care. Full-reference IQA (FR-IQA) methods, such as peak signal-to-noise ratio (PSNR) and structural similarity (SSIM), are often used to evaluate imaging conditions, reconstruction conditions, and image processing algorithms, including noise reduction and super-resolution technology. However, these IQA methods may be inapplicable for medical images because they were designed for natural images. Therefore, this study aimed to investigate the correlation between objective assessment by some FR-IQA methods and human subjective assessment for computed tomography (CT) images. For evaluation, 210 distorted images were created from six original images using two types of degradation: noise and blur. We employed nine widely used FR-IQA methods for natural images: PSNR, SSIM, feature similarity (FSIM), information fidelity criterion (IFC), visual information fidelity (VIF), noise quality measure (NQM), visual signal-to-noise ratio (VSNR), multi-scale SSIM (MSSSIM), and information content-weighted SSIM (IWSSIM). Six observers performed subjective assessments using the double stimulus continuous quality scale (DSCQS) method. The performance of IQA methods was quantified using Pearson’s linear correlation coefficient (PLCC), Spearman rank order correlation coefficient (SROCC), and root-mean-square error (RMSE). Nine FR-IQA methods developed for natural images were all strongly correlated with the subjective assessment (PLCC and SROCC > 0.8), indicating that these methods can apply to CT images. Particularly, VIF had the best values for all three items, PLCC, SROCC, and RMSE. These results suggest that VIF provides the most accurate alternative measure to subjective assessments for CT images.

## Introduction

The quality of medical images can vary due to complex relationships between various factors, such as equipment used, patient conditions, imaging conditions, and image reconstruction conditions, even when the same object is taken. Lesions may be overlooked if medical images have poor quality, and an accurate diagnosis would not be made, which is detrimental to patients. Therefore, proper assessment of medical image quality is a critical task in providing appropriate medical care.

Image quality assessment (IQA) methods are roughly divided into two types: subjective and objective assessments. Subjective assessments mean that humans visually judge the superiority or inferiority of image quality. For instance, in the evaluation of specific protocols or techniques, subjective assessments are the most reliable and gold standard method for IQA because humans are the final image handlers [1–5]. However, subjective assessments are time- and labor-consuming [1, 3–8]. In contrast, objective assessments use calculations to determine image quality; thus, these methods can be used as an alternative to subjective assessments because they address the problems associated with them. Physical indices such as modulation transfer function (MTF) and noise power spectrum (NPS) are commonly used as objective assessment methods for medical image quality. MTF is an index for assessing the resolution characteristics of an imaging system, and NPS is an index for assessing the noise characteristics of an imaging system. These indexes are, by their nature, based on signals obtained from dedicated phantoms or structures under defined conditions; therefore, directly assessing the quality of images containing complex structures, such as actual clinical images, can be difficult.

In the field of natural images, a lot of research on IQA methods for complex structures has been conducted [8–15], and their performances have been compared [4–7, 16–20]. IQA is classified into three types: full-reference IQA (FR-IQA), reduced-reference IQA (RR-IQA), and no-reference IQA (NR-IQA) [4, 5, 10, 15, 18, 20]. FR-IQA calculates an image quality score by comparing a reference image with a distorted image. The quality of the distorted image can be assessed by using a non-distorted image as the reference image. RR-IQA uses partial information from a reference image to calculate an image quality score for a distorted image. NR-IQA does not use a reference image and instead calculates an image quality score based solely on a distorted image. An IQA method with good performance judges the image quality as high quality in cases where humans perceive it as good, and low quality in cases where humans perceive it as poor. In other words, a good IQA method is strongly correlated with subjective assessments. In general, FR-IQA tends to calculate a highly correlated score with subjective assessments because it uses more information than NR-IQA [4]. Therefore, FR-IQA is useful when a reference image with the same geometric position as the target image can be acquired.

In the medical imaging field, IQA methods developed for natural images, especially traditional FR-IQA methods such as peak signal-to-noise ratio (PSNR) and structural similarity (SSIM), are widely used to evaluate imaging conditions, reconstruction conditions, and image processing algorithms, including noise reduction and super-resolution technology [3, 21–30]. However, because these IQA methods were designed for natural images, they may be inappropriate for assessing medical images with different properties. It is important to verify the applicability of IQA methods developed for natural images to medical images. Several research groups have evaluated the adaptability of FR-IQA methods to magnetic resonance (MR) images [31–33]. These studies evaluated the correlation between several FR-IQA methods and subjective assessments of various distorted MR images. Renieblas et al. evaluated several SSIM-related methods for distorted X-ray and MR images [34]. Furthermore, Kumar et al. investigated whether PSNR and SSIM adequately represent human subjective assessments as evaluation indices for determining the degree of image compression in teleradiology [35]. Currently, these are the only studies that have evaluated the adaptability of FR-IQA methods to medical images.

One of the major roles of IQA in medical imaging is to minimize patient burden while ensuring a certain level of image quality. Computed tomography (CT) is a modality with a relatively higher exposure dose than other imaging examinations; therefore, the importance of IQA is high. Nevertheless, no research has extensively investigated the applicability of IQA methods to CT images with various types of distortions.

Therefore, this study aimed to investigate the correlation between objective assessment by existing FR-IQA methods and human subjective assessment of CT images and evaluate whether FR-IQA methods developed for natural images can apply to CT image quality assessment.

In light of the current widespread use of the FR-IQA methods for medical image quality assessment, it is crucial to determine the reliability of the results obtained from these methods and that has great clinical significance. This study provides valuable evidence in evaluating the trustworthiness of the FR-IQA methods. Furthermore, this research identifies specific methods that are particularly useful for assessing CT image quality, thereby contributing to the advancement of objective assessment techniques in the field.

## Materials and Methods

### CT Images

**Fig. 1:**
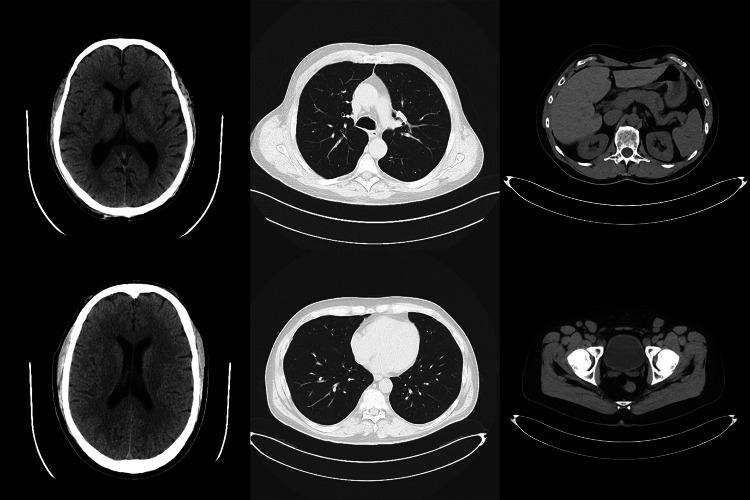
The six cases used as reference images

Six CT images were selected from open online databases as reference images, which were determined by a radiological technologist to have no significant artifacts and relatively low noise and blurring visually, and confirmed as such by another radiologist. The reference images included two images each of the head, chest, and abdomen (Fig. 1). The head images were obtained from CQ500 dataset [36], and the chest and abdominal images were obtained from DeepLesion dataset [37]. The CQ500 dataset provided by the Centre for Advanced Research in Imaging, Neurosciences, and Genomics (GARING) includes CT images of 491 individuals. The DeepLesion dataset published by the National Institutes of Health (NIH) contains CT images of various parts from the head to the legs of 4427 individuals. All the reference images obtained from both datasets were unsigned 16-bit portable network graphics (PNG) format, with image matrix size: 512 × 512 and slice thickness: 5 mm. In this study, we set the window width/window level to 80/40 Hounsfield units (HU), 1500/-650 HU, and 350/40 HU for the head, chest, and abdomen images, respectively, and then converted them linearly to 8-bit pixel values. The width/window level was determined in accordance with the clinical conditions actually used at Shiga University of Medical Science Hospital.

### Distorted Image Creation

**Fig. 2:**
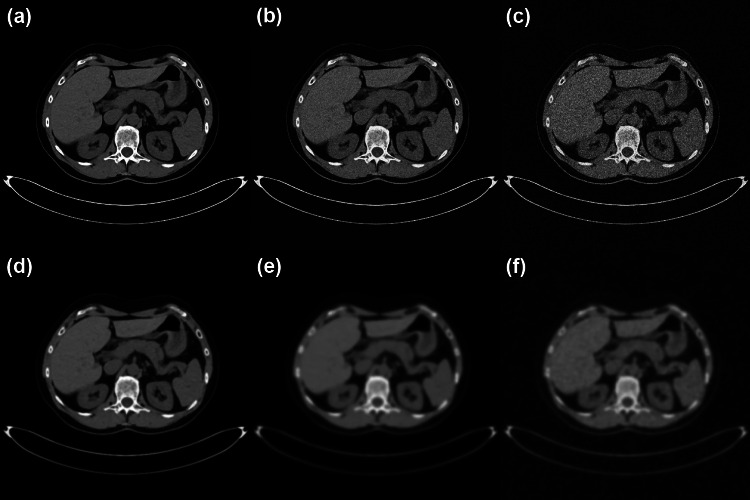
Examples of generated distorted images.** a** original image, **b** σ_noise_ = 14 and σ_blur_ = 0, **c** σ_noise_ = 30 and σ_blur_ = 0, **d** σ_noise_ = 0 and σ_blur_ = 1.4, **e** σ_noise_ = 0 and σ_blur_ = 3.0, **f** σ_noise_ = 30 and σ_blur_ = 3.0

The reference images were distorted using two types of distortion: Gaussian noise and Gaussian blur. In total, 210 distorted images were created by adding each or both types of distortion with five-grade intensity to the six reference images (Table 1 and Fig. 2). ImageJ software version 1.53e (Rasband W.S., Image J, U.S. National Institute of Health, Bethesda, Maryland, USA) was used to create the distorted images.

**Table 1:** Summary of distortions applied to reference images

Distortion type	Parameter	Intensity
Gaussian noise	Standard deviation	σ_noise_ = 6, 9, 14, 21, 30
Gaussian blur	Standard deviation	σ_blur_ = 0.6, 0.9, 1.4, 2.1, 3.0

### Objective Assessment

This study employed nine widely used FR-IQA methods for natural images: PSNR, SSIM [9], feature similarity (FSIM) [10], information fidelity criterion (IFC) [11], visual information fidelity (VIF) [8], noise quality measure (NQM) [12], visual signal-to-noise ratio (VSNR) [13], multi-scale SSIM (MSSSIM) [14], and information content-weighted SSIM (IWSSIM) [15]. PSNR is derived from mean squared error and indicates the ratio between the maximum pixel value and the power of the distortion. SSIM is a metric based on an assumption that human perception is highly adapted for extracting structural information from visual scenes and is calculated from three components: luminance, contrast, and structure. MSSSIM and IWSSIM were derived from SSIM. MSSSIM is calculated by weighting each component of SSIM on various scales. Consequently, MSSSIM is more flexible than SSIM in incorporating variations in viewing conditions. IWSSIM employs a weighted pooling strategy to estimate global image quality after local image quality measurements and uses the MSSSIM for local image quality measurements. FSIM is based on the fact that humans recognize an image mainly by its low-level features. FSIM computes quality estimates using phase congruency (PC) as the main feature and gradient magnitude (GM) as the complementary feature. IFC quantifies the amount of mutual information shared between the reference and distorted images. VIF is developed based on IFC and is calculated by normalizing IFC with reference image information. NQM is a human visual system (HVS) model-based index calculated by modeling the effect of additive noise on human perception systems. Similar to NQM, VSNR is an HVS model-based method. It is calculated based on both low-level and mid-level properties of human vision.

**Fig. 3:**
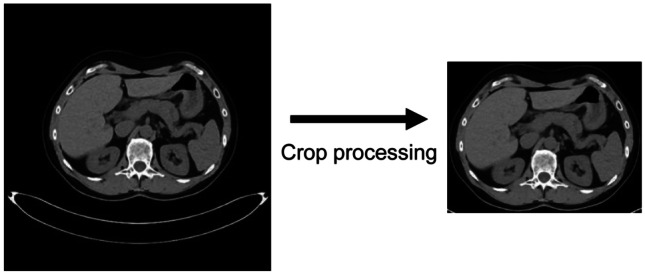
An example of cropping process to minimize background areas

In subjective assessments, the image quality of background areas with no diagnostic significance is expected to be almost ignored and not considered in the assessment, whereas, in objective assessments, the image quality of background areas affects the results to some extent. In this study, objective assessments were conducted on cropped images in which the background areas were minimized in order to purely focus on the distortion of the subject (Fig. 3).

### Subjective Assessment

**Fig. 4:**
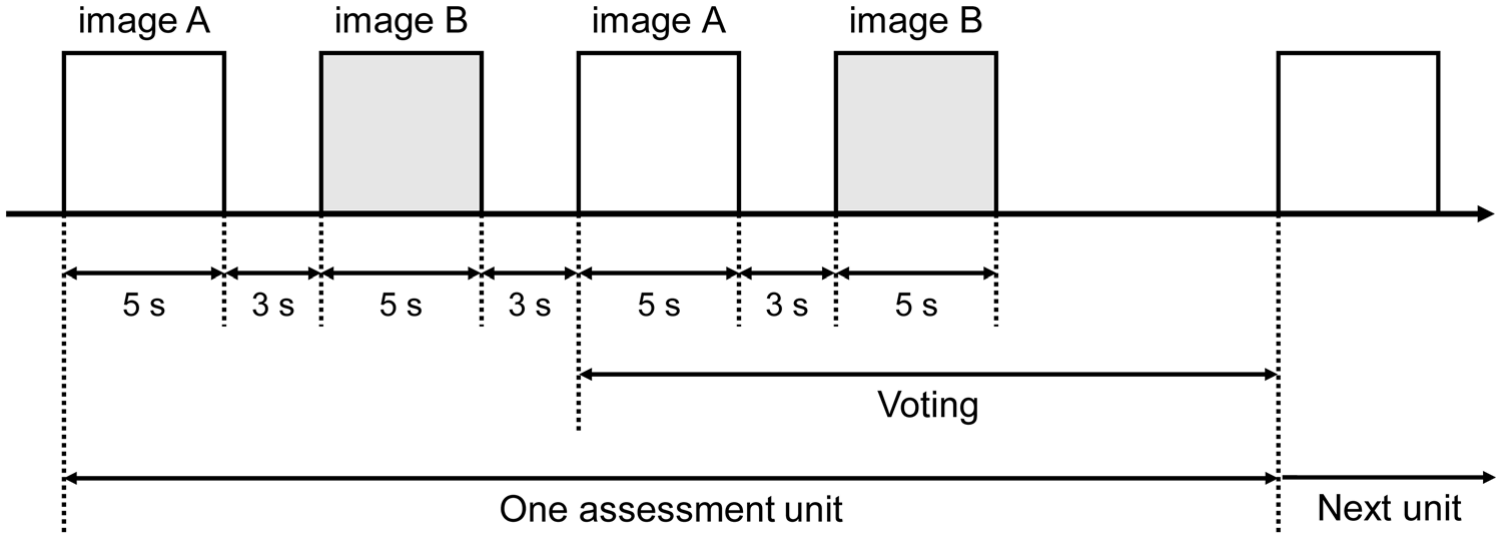
Assessment flow of the DSCQS method. Image A and B are a pair of reference and distorted images

**Fig. 5:**
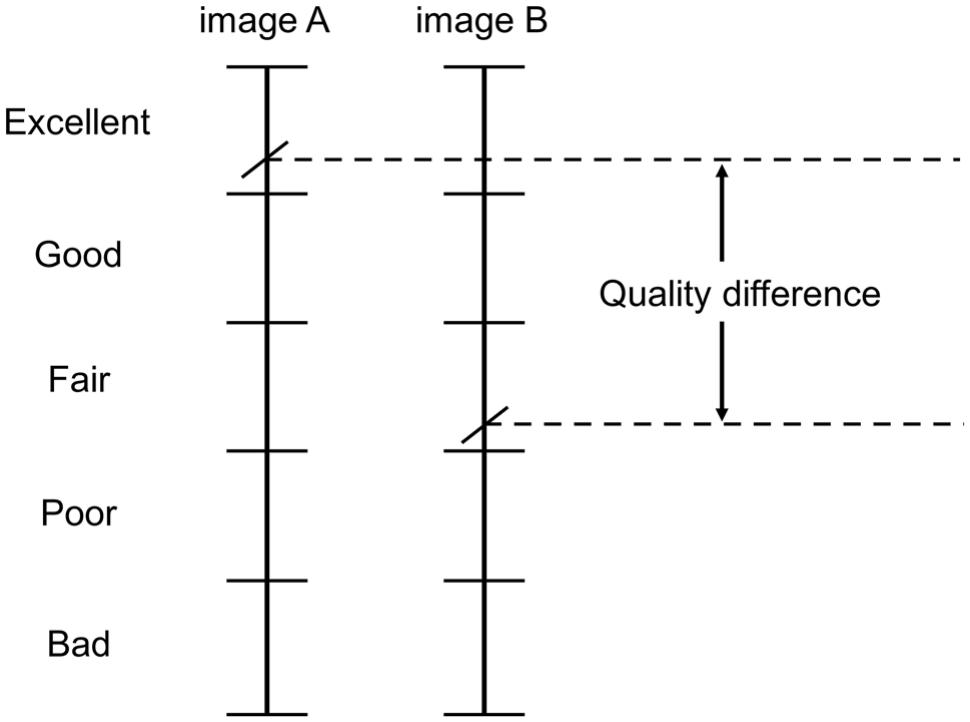
Rating scale used in the DSCQS method

Double stimulus continuous quality scale (DSCQS), a type of dual stimulation method proposed by the International Telecommunication Union Radiocommunication sector (ITU-R) Recommendation BT.500 [38], was used as the subjective assessment method. ITU-R BT.500 provides methodologies for the subjective assessment of image quality, including general testing methods, grading scales used during assessments, and viewing conditions recommended for carrying out assessments. Several IQA studies have performed subjective assessments based on this recommendation [5–7, 31, 39–42]. Figure 4 shows the assessment flow of the DSCQS method. Both reference and distorted images were presented in pairs alternately twice. An assessment score was assigned to both images at the second presentation. Observers were blind to which image was the reference image, and the display order for each image pair was random. The observers marked quality scores for both images on a continuous scale (Fig. 5). The marked quality scores were then normalized from 0 to 100, and the difference between the quality scores for the pair of images was calculated. The DSCQS score was calculated by averaging these quality differences for all the observers. The DSCQS score is derived from the quality difference; thus, a smaller value indicates better image quality, and a larger value indicates poorer image quality. Thanks to the dual-stimulus method and continuous scale, DSCQS is suitable for discriminating minute differences in image quality and is widely used in IQA for medical images [42–44]. However, because the reference and distorted images are presented twice separately, the assessment time is longer than for other subjective assessment methods. Here, we set the presentation time for each image to 5 s and the image display interval to 3 s.

For all FR-IQA methods used, higher and lower values indicate better and poorer image quality, respectively, which is inversely related to the DSCQS score. To interpret the results intuitively and easily, we defined the subjective score using the following formula:

1 $$Subjective\; Score=100-DSCQS\; Score.$$

Four radiologists and two radiological technologists were involved in the subjective assessment. Regarding the viewing conditions, the monitor was a two-megapixel medical monitor (RadiForce RX250, EIZO Corporation, Ishikawa, Japan), the illumination surrounding the display was set to 50 lx or less, and the viewing distance was arbitrary. The order of the displayed image pairs was randomized for each observer. At the beginning of the assessment, a training session was conducted to stabilize the observer’s opinion, using another case different from the six cases used for the assessment.

To assess the inter-rater variability of the subjective assessment, the intraclass correlation coefficient (ICC) was employed. There are several types of ICCs, depending on their intended use. In this study, we used ICC(2,1), which is the case for absolute agreement and single measurements. ICC(2,1) is a commonly used measure to evaluate agreement among multiple observers. The ICC(2,1) values range from 0 to 1, where higher values indicate greater agreement among observers. ICC(2,1) was calculated using SPSS version 25 (IBM Corp., Chicago, IL, USA).

### Performance Evaluation of FR-IQA Methods

Pearson linear correlation coefficient (PLCC), Spearman rank order correlation coefficient (SROCC), and root-mean-square error (RMSE) are generally used to evaluate IQA performance in terms of the prediction accuracy, monotonicity, and consistency of the models, respectively [5, 8–11, 13–16, 18, 20, 31, 39, 41, 42, 45]. The scores predicted using FR-IQA methods often do not correlate linearly with subjective assessment scores. PLCC and RMSE can be applied to linear systems; therefore, the scores require correction to eliminate nonlinearity using logistic regression as a pre-processing step. Here, we used five-parameter logistic regression [4, 5, 8, 15, 16, 19, 42, 45], which regression function is given as:

2 $${Y}_{L}={\beta }_{1}\left(\frac{1}{2}-\frac{1}{1+exp\left({\beta }_{2}\left(Y-{\beta }_{3}\right)\right)}\right)+{\beta }_{4}Y+{\beta }_{5}$$

where *Y* is the image quality score calculated by each IQA method; *Y*_*L*_ is the image quality score after regression; and *β*_*1*_, *β*_*2*_, *β*_*3*_, *β*_*4*_, and *β*_*5*_ are the regression model parameters. After the regression process, PLCC, SROCC, and RMSE were calculated using the following equations:

3 $$PLCC\left(X,{Y}_{L}\right)=\frac{{\sum }_{i}^{n}\left({X}_{i}-\overline{X }\right){\sum }_{i}^{n}\left({Y}_{Li}-\overline{{Y }_{L}}\right)}{\sqrt{{\sum }_{i}^{n}{\left({X}_{i}-\overline{X }\right)}^{2}}\sqrt{{\sum }_{i}^{n}{\left({Y}_{Li}-\overline{{Y }_{L}}\right)}^{2}}}$$

4 $$SROCC\left(X,Y\right)=1-\frac{6\sum_{i=1}^{n}{d}_{i}^{2}}{n\left({n}^{2}-1\right)}$$

5 $$RMSE\left(X,{Y}_{L}\right)=\sqrt{\frac{{\sum }_{i}^{n}{\left({Y}_{Li}-{X}_{i}\right)}^{2}}{n}}$$

where *X* is the subjective assessment score, *d* is the difference between *X* and *Y*, and *n* is the total number of images used in the evaluation. In this study, we applied these three metrics to 210 distorted images for overall FR-IQA performance evaluation. In addition, these metrics were also applied to 30 noise-distorted images without additional blurs, another 30 blur-distorted images without additional noises, 70 head images, 70 chest images, and 70 abdominal images to evaluate the performance of the FR-IQA methods for specific types of distortion and specific regions.

We also conducted statistical testing to confirm which differences between IQM performances were statistically significant. In the field of IQA, the *F*-test is usually performed on the residuals between the IQM scores after the regression and the subjective scores [4, 16, 32, 41]. The null hypothesis is that the data in the two IQA residual vectors have the same variance and they are statistically indistinguishable. If the null hypothesis is rejected after performing a one-tailed test at 5% significance level, then the performance of the two IQA methods is statistically significantly different.

Finally, we compared the average computation time required to assess an image for each FR-IQA method. We used 210 distorted images of 512 × 512 pixels before cropping and the average value of five calculations to calculate time. The CPU of the PC was an Intel Core i5-10210U with 8.00 GB memory, and the software platform was MATLAB R2021b (The MathWorks, Inc., Natick, Massachusetts). The MATLAB source code for all FR-IQA methods, except PSNR and SSIM, was obtained from the original authors. We calculated PSNR and SSIM using the built-in functions of MATLAB.

## Results

### Subjective Assessment

**Fig. 6:**
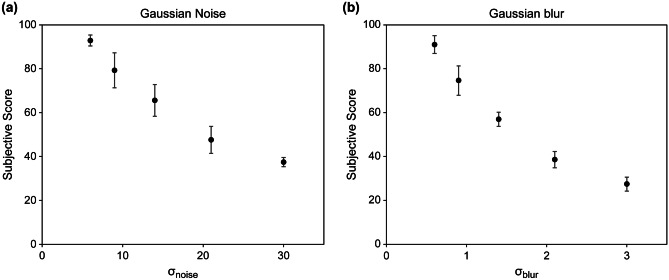
Results of subjective assessment for each distortion factor: **a** Gaussian noise and **b** Gaussian blur. Dots represent mean subjective scores of six observers; error bars represent standard deviation

Figure 6 shows the results of the subjective assessment of each distortion factor. The cases used in these results were independently distorted images that did not contain any other distortion factors. In other words, in Fig. 6a, only noise was added to the image, and the blur was fixed at σ_blur_ = 0. In Fig. 6b, only blur was added, and the noise was fixed at σ_noise_ = 0. The assessment results were obtained for the data of 30 cases because five levels of distortion intensity were applied to the six cases. The subjective score decreased as the distortion intensity increased for both the noise and blur. In addition, the rate of decrease became lower as the distortion intensity increased.

The obtained ICC(2,1) value for our subjective assessment was 0.68 (95% confidence interval: 0.61–0.74), indicating a moderate level of agreement among the observers. The 95% confidence interval suggests that the true ICC value falls between 0.61 and 0.74 with a high degree of certainty.

### IQA Performance Comparison

**Fig. 7:**
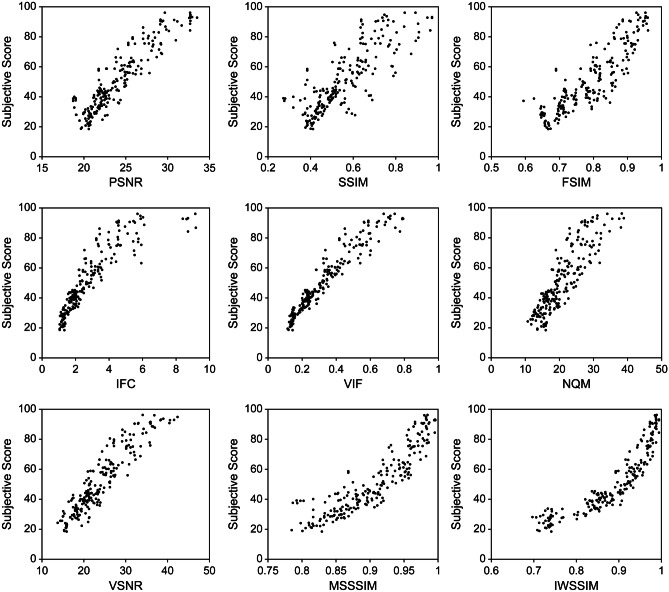
Scatter plots of the relationship between subjective scores and objective scores calculated by nine FR-IQA methods for 210 distorted images

Figure 7 shows scatter plots of the relationship between the objective scores calculated by each FR-IQA method and the subjective scores for the 210 distorted images. Table 2 compares the PLCC, SROCC, and RMSE results. All FR-IQA methods had PLCC and SROCC values higher than 0.8, indicating strong positive correlations. Particularly, VIF exhibited the best performance for all three indices: PLCC = 0.967, SROCC = 0.968, and RMSE = 5.380. In contrast, the SSIM was inferior to other FR-IQA methods: PLCC = 0.817, SROCC = 0.882, and RMSE = 12.075. Table 3 shows the statistical significance testing results among the nine FR-IQA methods for all 210 distorted images. The symbol “1” means that the method in the row is statistically better than the method in the column, the symbol “-” means that it is statistically indistinguishable, or the symbol “0” means that it is statistically worse. VIF and IWSSIM performed statistically better than the other IQM methods. In contrast, SSIM was statistically worse than any other IQM method.

**Table 2:** PLCC, SROCC, and RMSE values between subjective scores and objective scores by nine FR-IQA methods for 210 distorted images

	PSNR	SSIM	FSIM	IFC	VIF	NQM	VSNR	MSSSIM	IWSSIM
PLCC	0.918	0.817	0.881	0.944	**0.967**	0.878	0.928	0.931	0.960
SROCC	0.905	0.882	0.886	0.948	**0.968**	0.890	0.921	0.919	0.958
RMSE	8.303	12.075	9.912	6.913	**5.380**	10.023	7.831	7.631	5.874

The best performance values in each row are highlighted in boldface

**Table 3:** Statistical significance testing results based on prediction residuals of nine FR-IQA methods for all 210 distorted images. The symbol “1”, “-”, or “0” means that the method in the row is statistically (with 95% confidence) better, indistinguishable, or worse than the method in the column, respectively

	PSNR	SSIM	FSIM	IFC	VIF	NQM	VSNR	MSSSIM	IWSSIM
PSNR	-	1	1	0	0	1	-	-	0
SSIM	0	-	0	0	0	0	0	0	0
FSIM	0	1	-	0	0	-	0	0	0
IFC	1	1	1	-	0	1	-	-	0
VIF	1	1	1	1	-	1	1	1	-
NQM	0	1	-	0	0	-	0	0	0
VSNR	-	1	1	-	0	1	-	-	0
MSSSIM	-	1	1	-	0	1	-	-	0
IWSSIM	1	1	1	1	-	1	1	1	-

Table 4 compares the performance of the FR-IQA methods on 30 images degraded by each distortion type alone. Regarding noise, VIF had the best performance for PLCC and RMSE, and VSNR had the best performance for SROCC. Regarding blur, VIF had the best performance for PLCC and SROCC, and IWSSIM had the best performance for RMSE. Table 5 shows the statistical significance testing results among the nine FR-IQA methods for a particular distortion. Each entry in the table is a codeword consisting of two symbols. The symbol location within a codeword represents each type of distortion in the following order, from left to right: Gaussian noise and Gaussian blur. Regarding noise, IFC, VIF, VSNR, MSSSIM, and IWSSIM performed statistically better than the other four IQM methods. Regarding blur, VIF had the highest number of statistical superiority over the other methods and was statistically superior to PSNR, SSIM, IFC, NQM, and VSNR.

**Table 4:** PLCC, SROCC, and RMSE values between subjective scores and objective scores by nine FR-IQA methods for 30 distorted images by Gaussian noise without additional blur and another 30 distorted images by Gaussian blur without additional noise

		PSNR	SSIM	FSIM	IFC	VIF	NQM	VSNR	MSSSIM	IWSSIM
Noise	PLCC	0.958	0.854	0.854	0.959	**0.972**	0.896	0.971	0.966	0.963
	SROCC	0.918	0.851	0.887	0.939	0.970	0.912	**0.971**	0.959	0.951
	RMSE	10.351	17.547	12.953	6.632	**5.341**	10.014	6.890	10.960	6.328
Blur	PLCC	0.945	0.858	0.975	0.964	**0.985**	0.964	0.934	0.966	0.982
	SROCC	0.961	0.847	0.964	0.946	**0.982**	0.961	0.934	0.964	0.969
	RMSE	8.099	16.471	6.790	8.431	4.937	9.477	8.506	7.945	**4.930**

The best performance values in each row are highlighted in boldface

**Table 5:** Statistical significance testing results based on prediction residuals of nine FR-IQA methods for 30 images degraded by each distortion type alone. Each entry in the table is a codeword consisting of two symbols. The symbol location within a codeword represents each type of distortion in the following order: [Gaussian noise, Gaussian blur]. The symbol “1”, “-”, or “0” means that the method in the row, for a particular distortion, is statistically (with 95% confidence) better, indistinguishable, or worse than the method in the column, respectively

	PSNR	SSIM	FSIM	IFC	VIF	NQM	VSNR	MSSSIM	IWSSIM
PSNR	- -	- 1	- -	0 -	0 0	- -	0 -	0 -	0 0
SSIM	- 0	- -	- 0	0 0	0 0	- 0	0 -	0 0	0 0
FSIM	- -	- 1	- -	0 -	0 -	- -	0 1	0 -	0 -
IFC	1 -	1 1	1 -	- -	- 0	1 -	- -	- -	- -
VIF	1 1	1 1	1 -	- 1	- -	1 1	- 1	- -	- -
NQM	- -	- 1	- -	0 -	0 0	- -	0 -	0 -	0 -
VSNR	1 -	1 -	1 0	- -	- 0	1 -	- -	- -	- 0
MSSSIM	1 -	1 1	1 -	- -	- -	1 -	- -	- -	- -
IWSSIM	1 1	1 1	1 -	- -	- -	1 -	- 1	- -	- -

Table 6 compares the values of PLCC, SROCC, and RMSE for the head (70 images), chest (70 images), and abdomen (70 images). Regarding the head images, VIF had the best performance for PLCC and RMSE, and IWSSIM had the best performance for PLCC and SROCC. Regarding the chest images, VIF had the best performance for all indices, and IWSSIM also had the best performance for PLCC. Regarding the abdominal images, IWSSIM had the best performance for PLCC and SROCC, and VIF had the best performance for RMSE. Table 7 shows the statistical significance testing results among the nine FR-IQA methods for a particular target site. Each entry in the table is a codeword consisting of three symbols. The symbol location within a codeword represents each target site in the following order, from left to right: head, chest, and abdomen. Regarding head and abdomen, VIF and IWSSIM had the highest number of statistical superiority over the other methods and was statistically superior to PSNR, SSIM, FSIM, NQM, VSNR, and MSSIM. Regarding chest, VIF and IWSSIM also had the highest number of statistical superiority over the other methods and was statistically superior to PSNR, SSIM, FSIM, IFC, VSNR, and MSSIM.

**Table 6:** PLCC, SROCC, and RMSE values between subjective scores and objective scores by nine FR-IQA methods for 70 head images, 70 chest images, and 70 abdominal images

		PSNR	SSIM	FSIM	IFC	VIF	NQM	VSNR	MSSSIM	IWSSIM
Head	PLCC	0.931	0.762	0.932	0.957	**0.968**	0.899	0.939	0.913	**0.968**
	SROCC	0.942	0.788	0.926	0.958	0.953	0.932	0.949	0.907	**0.959**
	RMSE	9.840	15.247	11.689	7.909	**6.780**	11.317	7.800	9.259	7.505
Chest	PLCC	0.945	0.949	0.896	0.964	**0.982**	0.972	0.967	0.961	**0.982**
	SROCC	0.939	0.935	0.885	0.969	**0.985**	0.965	0.966	0.926	0.978
	RMSE	7.318	7.645	9.974	7.124	**4.519**	11.086	5.440	5.677	4.940
Abdomen	PLCC	0.938	0.795	0.943	0.961	0.976	0.959	0.884	0.933	**0.977**
	SROCC	0.897	0.784	0.945	0.953	0.969	0.963	0.857	0.912	**0.970**
	RMSE	7.513	12.104	7.655	5.484	**4.522**	7.101	9.672	7.532	4.771

The best performance values in each row are highlighted in boldface

**Table 7:** Statistical significance testing results based on prediction residuals of nine FR-IQA methods for 70 images by each target site. Each entry in the table is a codeword consisting of three symbols. The symbol location within a codeword represents each target site in the following order: [head, chest, abdomen]. The symbol “1”, “-”, or “0” means that the method in the row, for a particular target site, is statistically (with 95% confidence) better, indistinguishable, or worse than the method in the column, respectively

	PSNR	SSIM	FSIM	IFC	VIF	NQM	VSNR	MSSSIM	IWSSIM
PSNR	- - -	1 - 1	- 1 -	- - 0	0 0 0	- 0 -	- - 1	- - -	0 0 0
SSIM	0 - 0	- - -	0 1 0	0 - 0	0 0 0	0 0 0	0 - -	0 - 0	0 0 0
FSIM	- 0 -	1 0 1	- - -	- 0 -	0 0 0	- 0 -	- 0 1	- 0 -	0 0 0
IFC	- - 1	1 - 1	- 1 -	- - -	- 0 -	1 - -	- - 1	1 - 1	- 0 -
VIF	1 1 1	1 1 1	1 1 1	- 1 -	- - -	1 - 1	1 1 1	1 1 1	- - -
NQM	- 1 -	1 1 1	- 1 -	0 - -	0 - 0	- - -	0 - 1	- - -	0 - 0
VSNR	- - 0	1 - -	- 1 0	- - 0	0 0 0	1 - 0	- - -	- - 0	0 0 0
MSSSIM	- - -	1 - 1	- 1 -	0 - 0	0 0 0	- - -	- - 1	- - -	0 0 0
IWSSIM	1 1 1	1 1 1	1 1 1	- 1 -	- - -	1 - 1	1 1 1	1 1 1	- - -

Table 8 shows the average computation time required to calculate an image’s score using each FR-IQA method. PSNR had the shortest time (0.0041 s), while VIF had the longest (0.6183 s), with a difference of 0.6142 s.

**Table 8:** Average computation time per image for each FR-IQA method

Method	PSNR	SSIM	FSIM	IFC	VIF	NQM	VSNR	MSSSIM	IWSSIM
Time (seconds)	0.0041	0.0300	0.1590	0.6070	0.6183	0.1755	0.0772	0.0089	0.3208

## Discussion

In this study, subjective assessments using DSCQS method were used as the ground truth to evaluate the performance of the FR-IQA methods. We assessed the images distorted by two types of degradation factors: Gaussian noise and Gaussian blur. Gaussian noise makes low-contrast objects difficult to percept and degrades image quality in terms of graininess, whereas Gaussian blur obscures small objects and fine details and degrades image quality in terms of sharpness [31, 46]. The results in Fig. 6 reflect these facts and support that subjective assessments were properly conducted. In addition, as the intensity of distortion increased, the subjective rating decreased, and the rate of decrease progressively declined, approaching a plateau. We set the maximum distortion intensity values to σ_noise_ = 30 and σ_blur_ = 3.0. Considering this result, even if the distortion intensity was increased further, we expected no significant differences in the obtained subjective scores. Therefore, we considered that the distortion intensities used in this study cover a wide range of degradation that humans can perceive in medical images, and we believe that the settings of the distortion intensities were appropriate.

This study aimed to evaluate whether FR-IQA methods developed for natural images could be adapted to the quality assessment of medical images, such as CT images. Objective scores by all the nine FR-IQA methods used in this study had strong positive correlations (PLCC and SROCC) with the subjective assessment scores (Table 2). This suggests that FR-IQA methods developed for natural images can be used to assess the quality of CT images. Among the nine FR-IQA methods, VIF had the best performance, with the highest PLCC and SROCC and the lowest RMSE (Table 2). In statistical significance testing, VIF was also statistically better than other FR-IQA methods except for IWSSIM (Table 3). These results indicate that VIF is one of the most accurate methods for estimating CT image quality as a surrogate for subjective assessments. VIF was also shown to perform best in a previous study comparing subjective assessment with FR-IQA for MR images [32]. Furthermore, in the field of natural images, several studies have reported that VIF has superior performance for some datasets [6, 16, 17], particularly for multiple distortion databases [4]. Hence, we think that VIF is a method that expresses human perception well in the entire imaging field including all kinds of medical and natural images. Excluding IFC, VIF is unique among the nine IQA methods as it calculates a quality score based on the mutual information between a reference and a distorted image. IFC, a mutual information-based method like VIF, had the third highest correlation coefficient and third lowest RMSE among the nine FR-IQA methods. These approaches, based on mutual information, are considered useful in FR-IQA. Unlike IFC, VIF performs normalization based on reference image information, suppressing content dependence. This could explain why VIF outperformed IFC.

SSIM, now widely used to assess medical images, showed the lowest performance (Table 2). This is similar to previous studies that targeted MR images [32]. MSSSIM, an index derived from SSIM, showed performance improvement compared to SSIM. While SSIM is a single-scale approach, MSSSIM is a multi-scale approach. Based on the assumption that the optimal scale depends on conditions such as resolution and the distance between the image and the viewing point, MSSSIM is calculated by repeatedly low-pass filtering and down-sampling image pairs and weighting each component on various scales. This suggests that the single scale used in SSIM may not be optimal for this study’s image resolution and observation distance conditions. Moreover, among the SSIM-based methods used, IWSSIM performed the best. Both SSIM and MSSSIM employ a method that averages the scores calculated from each pixel to estimate global image quality, assuming each pixel has the same importance. In contrast, IWSSIM employs an information content-weighting strategy to estimate global image quality, which calculates weighted values for regions that humans are likely to pay attention to perceptually. Incidentally, IWSSIM uses MSSSIM for local quality estimation, which is also flexible to scale variations as MSSSIM. IWSSIM showed the second-best performance among the nine FR-IQA methods (Table 2) and was statistically better than other FR-IQA methods, similar to VIF (Table 3). This indicates that the pooling strategy used in IWSSIM performed excellently. Therefore, if SSIM-based methods are used, IWSSIM is the best choice because it is more flexible for scale and calculates a weighted global score.

In this study, even when the data were evaluated by dividing each distortion type and target site (Tables 4, 5, 6, and 7), VIF and IWSSIM, which demonstrated high performance in the overall evaluation results, showed consistently high performance and little bias due to distortion types or target sites.

In previous studies, FR-IQA methods for medical images evaluated single distorted images by each of several distortion types [31–35]. However, actual image distortion is caused by a mixture of various factors. In this study, we used multiple distorted images, including noise and blur, and achieved an evaluation of more complex distortion. Therefore, we believe that this study’s results can be applicable to more clinically realistic situations.

The biggest disadvantage of the FR-IQA method is that the image quality is evaluated using a reference image, which may be difficult to obtain in clinical situations. Techniques for directly evaluating clinical CT image quality belong to the NR-IQA category, and research in this category is also active. However, since the FR-IQA method mainly calculates the score from some kind of difference or similarity between the evaluation image and the reference image, it can be used in a wide range of applications regardless of the type of deterioration or target part. Therefore, FR-IQA is frequently used to evaluate imaging conditions, reconstruction conditions, and image processing algorithms, including noise reduction and super-resolution technology. However, since these indices were originally developed for natural images, this study’s motivation was to verify whether these indices can also be used to evaluate medical images. The results obtained in this study will support the reliability of the results of studies using the FR-IQA in the evaluation of CT image quality in the past.

**Fig. 8:**
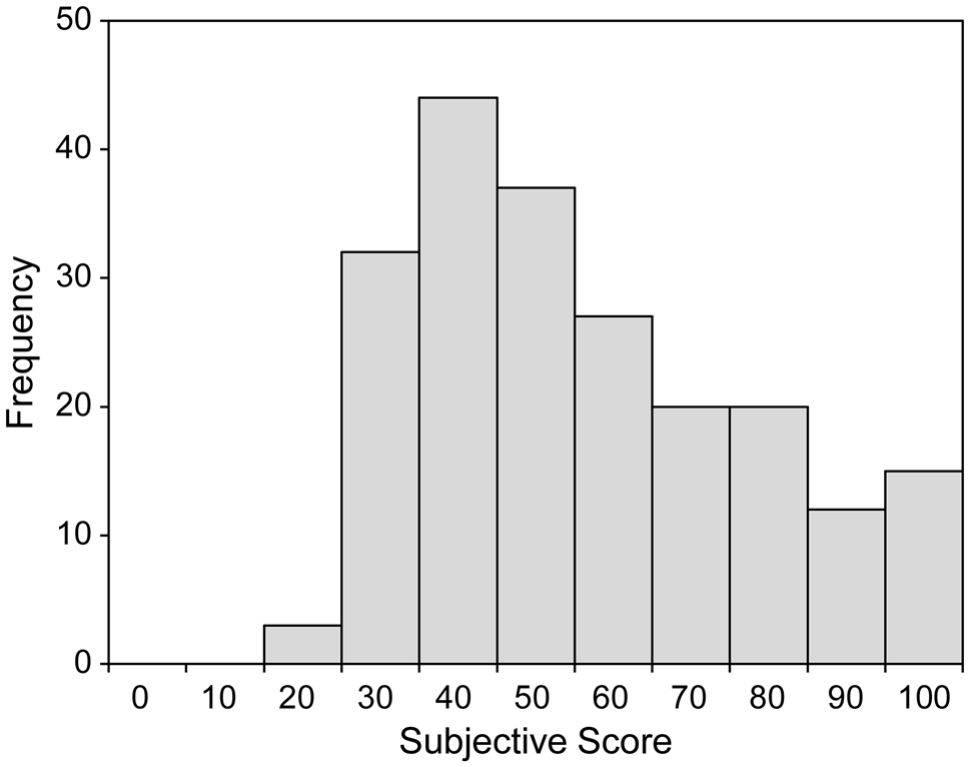
Histogram of subjective assessment scores for 210 distorted images

This study had several limitations. First, some FR-IQA methods require the setting of several parameters. We set the parameters for each FR-IQA method based on the values recommended in the original papers. Optimizing these parameters for medical images may improve the performance of the FR-IQA methods because these recommended values are for natural images. Second, the distribution of subjective assessment scores for the 210 distorted images was uneven. Figure 8 depicts a histogram of the subjective assessment scores. The volume zone was approximately 40, and the distribution was not uniform. Therefore, our results may have been strongly influenced by the distorted images near the volume zone. Finally, the distorted images used in this study were simulated artificially and may differ from actual clinical images. The noise in CT images follows a Gaussian distribution [42]; therefore, we adopted Gaussian noise. Unlike artificial distortions, clinical images do not necessarily exhibit uniform degradation throughout the entire image, and the degree of distortion may vary depending on the local area. Realistic distorted images are necessary; however, preparing images containing inhomogeneous various degrees of known degradations is difficult.

## Conclusion

The nine FR-IQA methods demonstrated excellent correlations with subjective assessment, suggesting that FR-IQA methods developed for natural images can be applied to CT images, and will support the reliability of the results of studies using the FR-IQA in the evaluation of CT image quality in the past. In particular, VIF demonstrated the highest performance and was the most useful method for assessing the quality of CT images.

## Data Availability

The DeepLesion dataset is available at https://nihcc.box.com/v/DeepLesion, and the CQ500 dataset is available at http://headctstudy.qure.ai/dataset.

## References

[1] Sui L, Ji L, Yongbo W, Yuting L, Dong Z, Zhaoying Z, Jianhua M: Blind CT image quality assessment via deep learning strategy: Initial Study. Proc SPIE 10577, 2018.

[2] Gao Q, Li S, Zhu M, Li D, Bian Z, Lv Q, Zeng D, Ma J (2020). Combined global and local information for blind CT image quality assessment via deep learning. Proc SPIE.

[3] Mudeng V, Kim M, Choe S (2022). Prospects of structural similarity index for medical image analysis. Appli Sci.

[4] Athar S, Wang Z (2019). A Comprehensive performance evaluation of image quality assessment algorithms. IEEE Access.

[5] Mohammadi P, Ebrahimi-Moghadam A, Shirani S (2015). Subjective and objective quality assessment of image: A survey. Majlesi J Electr Eng.

[6] Lin W, Kuo CC (2011). Perceptual visual quality metrics: A survey. J Vis Commun Image Represent.

[7] Ponomarenko N, Jin L, Ieremeiev O, Lukin V, Egiazarian K, Astola J, Vozel B, Chehdi K, Carli M, Battisti F, Kuo CC (2015). Image database TID2013: Peculiarities, results and perspectives. Signal Process Image Commun.

[8] Sheikh HR, Bovik AC (2006). Image information and visual quality. IEEE Trans Image Process.

[9] Wang Z, Bovik AC, Sheikh HR, Simoncelli EP (2004). Image quality assessment: from error visibility to structural similarity. IEEE Trans Image Process.

[10] Zhang L, Zhang L, Mou X, Zhang D (2011). FSIM: a feature similarity index for image quality assessment. IEEE Trans Image Process.

[11] Sheikh HR, Bovik AC, de Veciana G (2005). An information fidelity criterion for image quality assessment using natural scene statistics. IEEE Trans Image Process.

[12] Damera-Venkata N, Kite TD, Geisler WS, Evans BL, Bovik AC (2000). Image quality assessment based on a degradation model. IEEE Trans Image Process.

[13] Chandler DM, Hemami SS (2007). VSNR: a wavelet-based visual signal-to-noise ratio for natural images. IEEE Trans Image Process.

[14] Wang Z, Simoncelli EP, Bovik AC: Multiscale structural similarity for image quality assessment. Proc. 37th IEEE Asilomar Conference on Signals, Systems and Computers, 2003.

[15] Wang Z, Li Q (2011). Information content weighting for perceptual image quality assessment. IEEE Trans Image Process.

[16] Sheikh HR, Sabir MF, Bovik AC (2006). A statistical evaluation of recent full reference image quality assessment algorithms. IEEE Trans Image Process.

[17] Pedersen M, Hardeberg JY (2012). Full-reference image quality metrics: classification and evaluation. Found Trends in Comput Graph Vis.

[18] Zhang L, Mou X, Zhang D: A comprehensive evaluation of full reference image quality assessment algorithms. IEEE Int Conf Image Processing. 1477–1480, 2012.

[19] Pedersen M: Evaluation of 60 full-reference image quality metrics on the CID: IQ2015. IEEE Int Conf Image Processing 1588–1592, 2015.

[20] Niu Y, Zhong Y, Guo W, Shi Y (2019). ChenP: 2D and 3D image quality assessment: A survey of metrics and challenges. IEEE Access.

[21] Jadick G, Abadi E, Harrawood B, Sharma S, Segars WP, Samei E: A framework to simulate CT images with tube current modulation. SPIE Med Imaging 11595, 2021. 10.1088/1361-6560/ac2269 PMC8552241 34464942

[22] Joemai RMS, Geleijns J (2017). Assessment of structural similarity in CT using filtered backprojection and iterative reconstruction: a phantom study with 3D printed lung vessels. Br J Radiol.

[23] Park HJ, Choi SY, Lee JE, Lim S, Lee MH, Yi BH, Cha JG, Min JH, Lee B, Jung Y (2022). Deep learning image reconstruction algorithm for abdominal multidetector CT at different tube voltages: assessment of image quality and radiation dose in a phantom study. Eur Radiol.

[24] Knoll F, Hammernik K, Zhang C, Moeller S, Pock T, Sodickson DK, Akçakaya M (2020). Deep-Learning Methods for Parallel Magnetic Resonance Imaging Reconstruction: A Survey of the Current Approaches, Trends, and Issues. IEEE Signal Process Mag.

[25] Han M, Shim H, Baek J (2021). Low-dose CT denoising via convolutional neural network with an observer loss function. Med Phys.

[26] Kidoh M, Shinoda K, Kitajima M, Isogawa K, Nambu M, Uetani H, Morita K, Nakaura T, Tateishi M, Yamashita Y, Yamashita Y (2020). Deep learning based noise reduction for brain MR imaging: Tests on phantoms and healthy volunteers. Magn Reson Med Sci.

[27] Jin Y, Jiang XB, Wei ZK (2019). LiY: Chest X-ray image denoising method based on deep convolution neural network. IET Image Process.

[28] Umehara K, Ota J, Ishida T (2018). Application of Super-Resolution Convolutional Neural Network for Enhancing Image Resolution in Chest CT. J Digit Imaging.

[29] Wang J, Chen Y, Wu Y, Shi J, Gee J: Enhanced generative adversarial network for 3D brain MRI super-resolution. IEEE Winter Conf Appl Comput Vis 3627–3636, 2020.

[30] Umehara K, Junko O, Naoki I, Shunsuke O, Kentaro O, Takanori S, Naoki S, Takayuki I: Super-resolution convolutional neural network for the improvement of the image quality of magnified images in chest radiographs. Proceedings of the SPIE 10133:101331P-1 - 101331P-7, 2017.

[31] Chow LS, Rajagopal H, Paramesran R (2016). Correlation between subjective and objective assessment of magnetic resonance (MR) images. Magn Reson Imaging.

[32] Mason A, Rioux J, Clarke SE, Costa A, Schmidt M, Keough V, Huynh T, Beyea S (2020). Comparison of objective image quality metrics to expert radiologists’ scoring of diagnostic quality of MR images. IEEE Trans Med Imaging.

[33] Kastryulin S, Zakirov J, Pezzotti N, Dylov DV (2023). Image quality assessment for magnetic resonance imaging. IEEE Access.

[34] Renieblas GP, Nogués AT, González AM, Gómez-Leon N, Del Castillo EG (2017). Structural similarity index family for image quality assessment in radiological images. J Med Imaging (Bellingham).

[35] Kumar B, Singh SP, Mohan A, Singh HV: MOS prediction of SPIHT medical images using objective quality parameters. Int Conf Signal Process Systems 219–223, 2009.

[36] Chilamkurthy S, Ghosh R, Tanamala S, Biviji M, Campeau NG, Venugopal VK, Mahajan V, Rao P, Warier P (2018). Deep learning algorithms for detection of critical findings in head CT scans: a retrospective study. Lancet.

[37] Yan K, Wang X, Lu L, Summers RM (2018). DeepLesion: automated mining of large-scale lesion annotations and universal lesion detection with deep learning. J Med Imaging (Bellingham).

[38] Recommendation ITU-R BT. 500–14: Methodologies for the subjective assessment of the quality of television images. 2019.

[39] Corchs S, Gasparini F, Schettini R: Noisy images-JPEG compressed: subjective and objective image quality evaluation. Proc SPIE 9016:90160V-1 – 90160V-9, 2014.

[40] Jayaraman D, Mittal A, Moorthy AK, Bovik AC: Objective quality assessment of multiply distorted images. Conf Rec Asilomar Conf 1693–1697, 2012.

[41] Gu K, Zhai G, Yang X, Zhang W (2014). Hybrid no-reference quality metric for singly and multiply distorted images. IEEE Trans Broadcast.

[42] Chow LS, Paramesran R (2016). Review of medical image quality assessment. Biomed Signal Process Control.

[43] Lévêque L, Outtas M, Liu H, Zhang L: Comparative study of the methodologies used for subjective medical image quality assessment. Phys Med Biol 66(15):2021. 10.1088/1361-6560/ac1157 34225264

[44] Lévêque L, Liu H, Barakovic S, Barakovic J, Martini M, Outtas M, Zhang L, Kumcu A, Platisa L, Rodrigues R, Pinheiro A, Skodras A: On the subjective assessment of the perceived quality of medical images and videos. Int Conf Qual Multimed Exp (QoMEX), 2018.

[45] Sun W, Zhou F (2017). LiaoQ; MDID: A multiply distorted image database for image quality assessment. Pattern Recognit.

[46] Sprawls P (1992). AAPM tutorial. CT image detail and noise. Radiographics.

